# GLUT2/SLC2A2 is a bi-directional urate transporter

**DOI:** 10.1016/j.jbc.2025.108485

**Published:** 2025-04-08

**Authors:** Yu Toyoda, Ryuichiro Shigesawa, Tony R. Merriman, Hirotaka Matsuo, Tappei Takada

**Affiliations:** 1 Department of Integrative Physiology and Bio-Nano Medicine, National Defense Medical College, Tokorozawa, Saitama, Japan; 2 Department of Pharmacy, The University of Tokyo Hospital, Bunkyo, Tokyo, Japan; 3 Division of Clinical Immunology and Rheumatology, University of Alabama at Birmingham, Birmingham, Alabama, USA

**Keywords:** uric acid, GWAS, urate handling, urate importer, urate exporter

## Abstract

Recent genetic studies showed an association between *solute carrier 2A2* (*SLC2A2*), which encodes glucose transporter 2 (GLUT2), and serum urate concentrations; however, urate transport activity of GLUT2 has not been studied contrary to its function as a sugar transporter. Here, we hypothesized that GLUT2 acts also as a urate transporter, which led us to conduct cell-based functional analyses using HEK-derived 293A cells. We found that radiolabeled [8-^14^C]-urate was incorporated into GLUT2-expressing cells more compared to control cells and this elevated cellular activity was almost completely inhibited by GLUT2 inhibitors, demonstrating that GLUT2 is a urate transporter. Regarding the concentration dependence of GLUT2-mediated urate transport, no saturable properties were observed within an experimentally achievable range (0–500 μM), suggesting that GLUT2 mediates the robust transport of urate. Moreover, the GLUT2-mediated urate transport was not inhibited by 10 mM glucose; GLUT2-mediated sugar transport was hardly affected by 500 μM urate. As these concentrations of urate and glucose were relevant to their maximum levels in healthy humans, our results suggest that GLUT2 maintains its urate transport ability under physiological conditions. Furthermore, using a cell-based urate efflux assay system, we successfully demonstrated that urate secretion was accelerated in GLUT2-expressing cells than in control cells. Therefore, GLUT2 may also function as a urate exporter. The present study revealed that GLUT2 is a bi-directional urate transporter. Our findings contribute to a deeper understanding of urate-handling systems in the body. To elucidate the physiological role of GLUT2 as a urate transporter, further studies are required.

Elevated serum urate concentrations can cause gout, which is an important public health issue and the most common type of inflammatory arthritis. To elucidate the genetic factors influencing serum urate levels, large-scale genome-wide association studies (GWASs) have been conducted, which revealed gout risk- and/or serum urate-associated genetic variants at numerous loci (1, 2). Such investigations have led to the discovery of molecular machineries regulating urate behavior, including physiologically important urate transporters; however, the causal mechanisms of the association between the identified variants and serum urate remain unclear in many genes.

Since urate—the primary form of uric acid under physiological conditions—cannot passively penetrate the cellular membrane owing to its hydrophilic properties and is the final metabolite in human purine metabolism, urate transporters play pivotal roles in urate handling, especially in humans (3). Considering that previously identified urate transporters alone cannot explain the complexity of urate transport, the presence of latent, physiologically important urate transporters has been suggested. Thus, it has attracted widespread interest to address whether membrane proteins encoded by genes, whose association with gout and/or serum urate was genetically found, can transport urate or not.

We have recently identified significant signals associated with gout located on *solute carrier 2A2* (*SLC2A2*) (Fig. S1) (1), which is consistent with the results of another large-scale study showing an association between *SLC2A2* and serum urate (4); the details are to be mentioned in a later section. *SLC2A2*, also known as glucose transporter 2 (GLUT2), encodes a physiologically important sugar transporter that prefers dietary sugars, including glucose and fructose, and is involved in their regulation in various organs such as the liver, kidney, and small intestine (5, 6, 7). However, to the best of our knowledge, the urate transport activity of GLUT2 has not been studied.

In humans, *SLC2A* genes encode 14 GLUT proteins, which are classified into three classes based on sequence homology: Class I (GLUT1–4, and 14), Class II (5, 7, 9, and 11), and Class III (6, 8, 10, 12, and 13) (6). Among them, GLUT9 (8, 9) and GLUT12 (10) have been identified as urate transporters of which dysfunction influences serum urate. In general, overlapping substrate specificities are often observed among transporters that are classified into the same category (a protein family or a further subdivided phylogenetically distinct class). For instance, in the SLC22A family, to which the first identified physiologically important urate transporter—urate transporter 1 (URAT1)/SLC22A12 (11)—belongs, other urate transporters have been identified including organic anion transporter 10 (OAT10)/SLC22A13 (12, 13). Based on these pieces of information, we herein hypothesized that GLUT2 can transport urate as well as sugars and experimentally tested this hypothesis.

## Results

### Functional expression of GLUT2 as a sugar transporter

**Figure 1:**
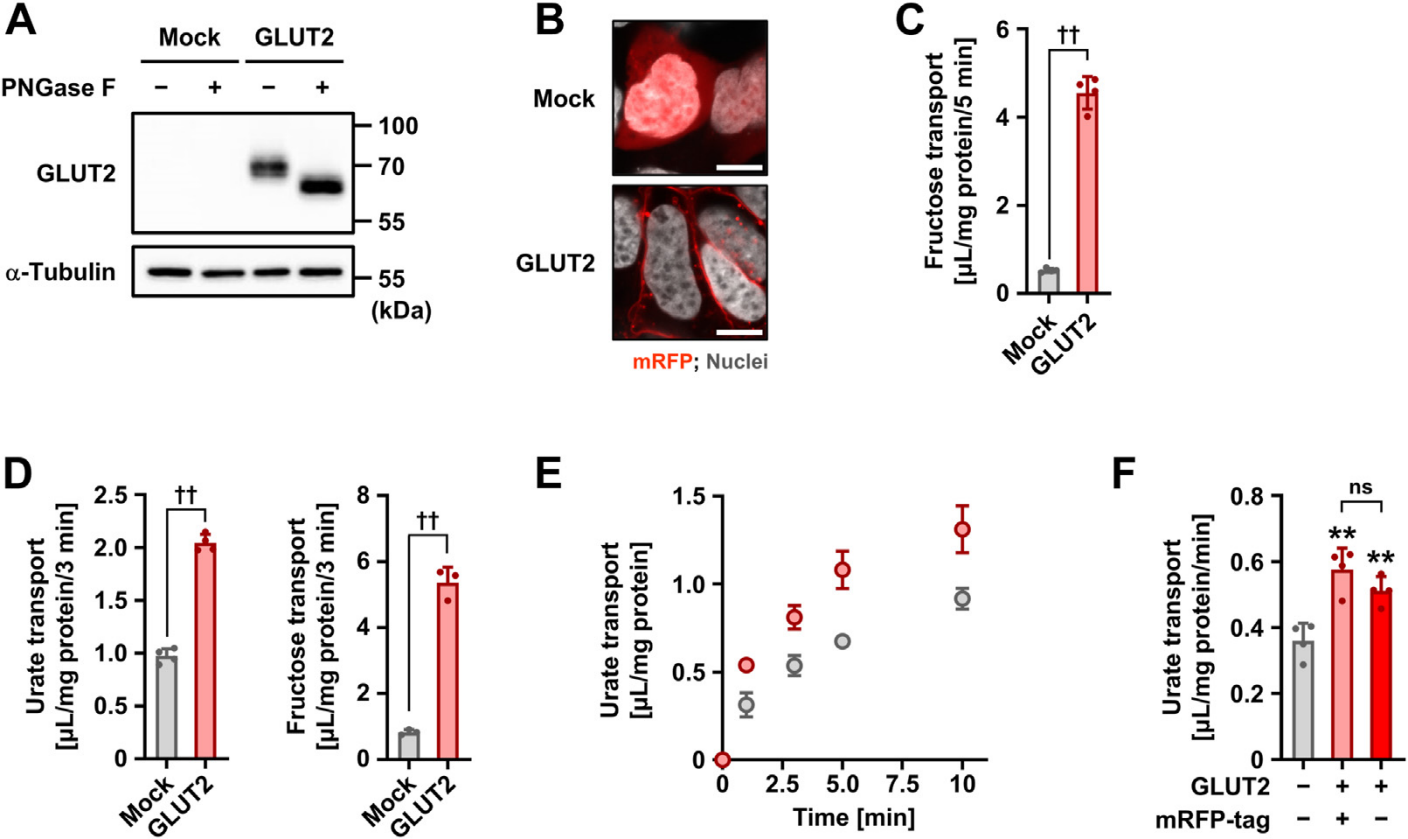
**Functional expression and urate transport activity of GLUT2**. We used 293A cells transiently expressing GLUT2 48 h after plasmid transfection. *A*, immunoblot detection of GLUT2 in whole-cell lysates. α-Tubulin, a loading control; mock, control transfected with empty vector (pmRFP-C1 plasmid without insert). *B*, intracellular localization of GLUT2 detected using confocal microscopy. Scale bars, 10 μm. *C*, functional expression of GLUT2 evidenced by [^3^H(G)]-fructose transport into the cells. *D*, elevated urate transport activity in GLUT2-expressing cells. At pH 6.4, cellular transport activities for [8-^14^C]-urate (*left*) and [^3^H(G)]-fructose (*right*) were examined. *E*, time-dependent [8-^14^C]-urate uptake by GLUT2-expressing or mock cells. *F*, mRFP tag does not significantly affect the urate transport activity of GLUT2. Transport assays were conducted using glucose- and glutamine-free Krebs–Ringer buffer at pH 7.4 (*C*, *E*, and *F*) or pH 6.4 (*D*). The concentrations of [8-^14^C]-urate and [^3^H(G)]-fructose in the transport buffer were 10 μM and 100 nM, respectively. Incubation time: 5 min (*C*), 3 min (*D*), 1 min (*F*). Data are expressed as the mean ± SD; *n* = 3 to 4 (*D*), 4 (*C*, *E*, and *F*). ††*p* < 0.01 (two-sided *t* test in *C* and *D*); ∗∗*p* < 0.01 *versus* mock; ns, not significantly different between indicated groups (Tukey–Kramer multiple-comparison test in *F*).

Prior to investigating the latent ability of GLUT2 to function as a urate transporter, we confirmed its functional expression in a cell-based assay system (Fig. 1). Forty-eight hours after plasmid transfection, the expression as a matured *N*-linked glycoprotein (Fig. 1*A*) and the plasma membrane localization (Fig. 1*B*) of mRFP-tagged GLUT2 (mRFP-GLUT2) were detected by immunoblotting and confocal microscopy, respectively. The GLUT2-mediated transport of fructose, a GLUT2 substrate (5), was also confirmed in cells incubated in glucose- and glutamine-free Krebs–Ringer buffer, which contained appropriate ionic components and buffering capacity (Fig. 1*C*).

### Identification of GLUT2 as a urate transporter

Next, we determined whether urate is a GLUT2 substrate. Based on previous studies (10, 14) which found that GLUT12—another SLC2A family protein—exhibited increased transport activities at pH conditions lower than 7.4, we used pH 6.4 in the first investigation. Similar to [^3^H(G)]-fructose, [8-^14^C]-urate was incorporated into GLUT2-expressing cells at higher levels than into mock cells, suggesting that GLUT2 is a urate transporter (Fig. 1*D*). Based on the results of the time-course experiment at pH 7.4 (Fig. 1*E*), we used urate uptake for 1 min for the evaluation of GLUT2-mediated transport in subsequent analyses. Also, the mRFP tag had little effect on the urate transport activity of GLUT2 (Fig. 1*F*).

### Characterization of GLUT2-mediated urate transport

**Figure 2:**
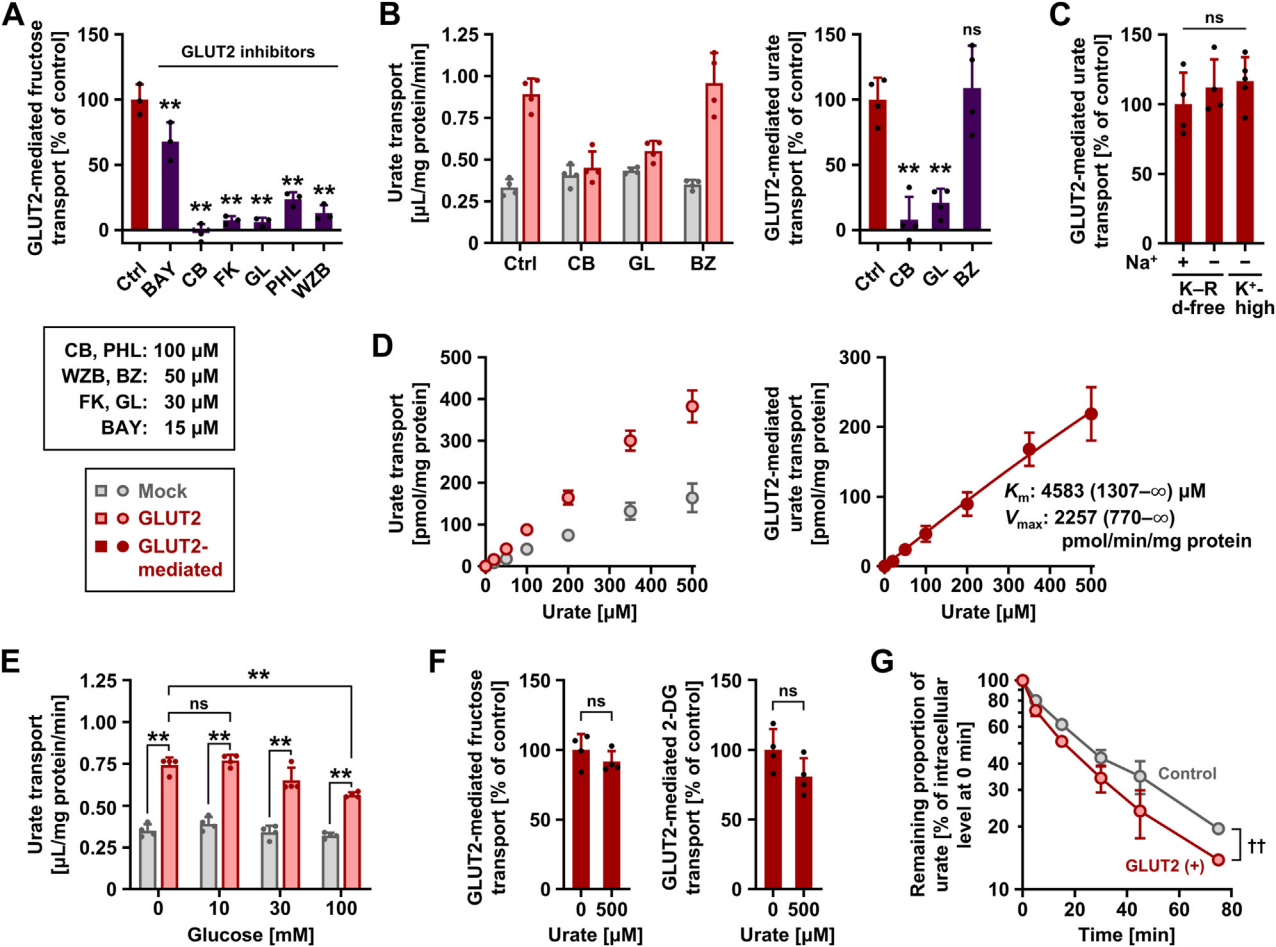
**Characterization of GLUT2 as a bi-directional urate transporter**. Uptake assays (*A*–*F*) were conducted in glucose- and glutamine-free Krebs–Ringer buffer (K–R d-free) using 293A cells transiently expressing GLUT2 48 h after plasmid transfection. Experimental conditions were as follows: 1 min incubation time and 10 μM [8-^14^C]-urate in the transport buffer (pH 7.4), unless otherwise specified; 100 nM [^3^H(G)]-fructose and 16.7 μM [1,2-^3^H(N)]-2-deoxy-D-glucose (2-DG) in the transport buffer; and 1 min incubation time for the evaluation of these sugar transporting activities. Urate efflux assay (*G*) was conducted as described in Supplementary information. Mock, empty vector (pmRFP-C1 plasmid without insert)-transfected controls. *A*, inhibitory effects of GLUT2 inhibitors on GLUT2-mediated fructose transport. Ctrl, vehicle control (0.5% dimethyl sulfoxide); BAY, BAY-876; CB, cytochalasin B; FK, forskolin; GL, glutor; PHL, phloretin; WZB, WZB117. *B*, effects of GLUT2 inhibitors (CB and GL) and benzbromarone (BZ) on the cellular (*left*) and GLUT2-mediated (*right*) urate transport activities. *C*, urate transport activity of GLUT2 in various transport buffers. *D*, investigation of concentration dependency. [8-^14^C]-Urate uptake by GLUT2-expressing or mock cells (*left*); GLUT2-mediated transport of [8-^14^C]-urate (*right*). The 95% confidence intervals are indicated in parentheses for the estimated values of the Michaelis–Menten constant (*K*_m_) and maximal velocity (*V*_max_). *E*, effects of glucose on cellular urate transport activities. The corresponding GLUT2-mediated urate transport activities are shown in Fig. S2. *F*, the minimal inhibitory effect of urate on GLUT2-mediated transport of [^3^H(G)]-fructose (*left*) and [1,2-^3^H(N)]-2-DG (*right*). *G*, time-dependent decrease in cellular [8-^14^C]-urate levels. Each value for the remaining proportion was calculated by subtracting the corresponding value of the secreted proportion at the indicated time points from 100 [%]. A base-10 logarithmic scale is used for the vertical axis. Data are expressed as the mean ± SD; where the vertical bars are not visible, the SD was contained within the limits of the symbol; *n* = 3 (*A*), 4 (*B*, *E*, *F*, and *G*), 4 to 5 (*C*), 6 (*D*). ∗∗*p* < 0.01 [Dunnett's test (*versus* control) in *A* and *B*; Tukey–Kramer multiple-comparison test in *C* and *E*]; ns, not significantly different between the indicated groups (two-sided *t* test in *F*). In (*G*), two-factor repeated measures ANOVA showed no significant group [control *versus* GLUT2 (+)] × time interaction (*p* = 0.067) and a significant effect of GLUT2 on the remaining proportion of urate (^††^*p* = 0.002).

To better understand the biochemical features of GLUT2 as a urate transporter, further analyses were performed in neutral pH conditions. To confirm the cellular urate transport activity originating from the introduction of GLUT2 was certainly mediated by GLUT2, we conducted an inhibition assay. Among commercially available GLUT2 inhibitors we tested, cytochalasin B (100 μM) and glutor (30 μM) showed good inhibitory effects on the GLUT2-mediated fructose transport activity (Fig. 2*A*); these two GLUT2 inhibitors exhibited similar results regarding the urate transport activity (Fig. 2*B*). Also, benzbromarone (50 μM)—an inhibitor for various urate transporters including URAT1, OAT10, ABCG2, and GLUT9 [IC_50_ values of benzbromarone against urate transport mediated by these transporters are reportedly less than 50 μM (12, 15, 16)]—hardly affected the difference in the urate transport activities between GLUT2-expressing and control cells, suggesting the minimal contribution of benzbromarone-sensitive urate transporters to the differences. These results indicate that the urate transport was mediated by GLUT2. Moreover, GLUT2-mediated urate uptake was not affected by the exclusion of sodium from the glucose- and glutamine-free Krebs–Ringer buffer, which mimics the ionic content of plasma, nor a high-potassium buffer condition that depolarizes plasma membranes, indicating sodium independence and bidirectionality of the urate transport function of GLUT2 (Fig. 2*C*).

GLUT2-mediated urate transport was not saturated (Fig. 2*D*) within the range of urate concentrations tested (0–500 μM; experimentally achievable concentrations due to the low solubility limitation of uric acid under neutral conditions), suggesting that GLUT2 mediates the robust transport of urate. The parameters calculated for GLUT2-mediated urate transport were Michaelis–Menten constant (*K*_m_), maximal velocity (*V*_max_), and R squared, which were 4583 μM, 2257 pmol/min/mg protein (Fig. 2*D*), and 0.95, respectively. Considering the physiological levels of serum urate in healthy humans (120–420 μM [approximately 2–7 mg/dl] (17)), the function of GLUT2 as a urate transporter would not be saturated in the human body, at least from the viewpoint of the substrate concentration.

Because glucose is a physiologically relevant substrate of GLUT2, we investigated its effects on GLUT2 function (Fig. 2*E*)—10 mM (approximately 180 mg/dl) glucose hardly affected the urate transport activity, while 100 mM glucose exhibited a significant inhibitory effect and decreased the GLUT2-mediated urate transport to approximately 60% of vehicle control (Fig. S2). Considering that blood glucose concentrations are generally <10 mM regardless of fasting or after eating, our results suggest that GLUT2-mediated urate transport might be hardly influenced by blood glucose. In addition, 10 mM glucose did not suppress the GLUT2-mediated fructose transport (Fig. S3). Moreover, we found that 500 μM urate had minimal inhibitory effects on GLUT2-mediated transport of fructose and 2-DG (Fig. 2*F*). These results suggest that GLUT2 can independently transport each substrate under physiological conditions, which is consistent with its unique characteristic of having low affinity for sugars (*K*_m_ for glucose, 17 mM; for fructose, 76 mM) compared with other glucose transporters (6).

### Urate efflux activity of GLUT2

Finally, we investigated whether GLUT2 exports urate from cells (Fig. S4), using a cell-based urate efflux assay system (18). In this system (Fig. S4*A*; details are described in Supplementary Methods), sufficient incorporation of [8-^14^C]-urate into cells was achieved by overexpressing SVCT2 as a urate uptake machinery, followed by the measurement of radioactivity secreted from the cells into fresh urate- and sodium-free culture medium in which SVCT2 cannot function. Notably, because human-derived 293A cells lack the urate-degrading enzyme uricase, which is not present in humans (19), intracellular urate is not metabolized in this assay system, allowing us to trace the [8-^14^C]-urate efflux.

In terms of ABCG2, a known urate exporter (20) that was used as a positive control, its co-transduction reduced the apparent urate uptake activity mediated by SVCT2 (Fig. S4*B*); the apparent urate efflux activities in ABCG2-expressing cells were significantly higher than those in control cells (Fig. S4*C*). These results are consistent with those of a previous study (18). In terms of GLUT2, although cellular [8-^14^C]-urate levels in GLUT2-expressing cells at the start of the secretion step (0 min) were significantly higher than those in control cells, those at the end of the assay (75 min) were significantly lower (Fig. S4*B*). Similarly, the apparent urate efflux activities in GLUT2-expressing cells were significantly higher than those in control cells (Fig. S4*C*). We then calculated the remaining proportion to the initial cellular amount of [8-^14^C]-urate, which was defined as follows: (1 − net amount of media-released [8-^14^C]-urate at indicated timepoint/initial amount of cellular [8-^14^C]-urate at 0 min) × 100 [%]. As expected, the time-dependent decrease in the remaining proportion of GLUT2-expressing cells was greater than that of control cells (Fig. 2*G*). The detection of accelerated urate secretion in GLUT2-expressing cells suggests that GLUT2 acts as a urate exporter.

## Discussion

In this study, we demonstrated the bidirectional urate transport activity of GLUT2 (a Class I member), which extends our understanding of both GLUT proteins and urate transporters. Given that GLUT9 (a Class II member) (8) and GLUT12 (a Class III member) (10) have physiological roles as urate transporters, our findings indicated that each class of the GLUT family contains at least one urate transporter, which also highlights that GLUT proteins are not necessarily specific only to sugars. In this context, some GLUT proteins, regardless of class, may have structural features for urate recognition as a common non-sugar substrate. Moreover, in relation to previous findings showing an association between genetic variations in *GLUT2*/*SLC2A2* and gout/serum urate (Table 1 and Fig. S1) (1, 4), our findings here suggest that GLUT2 might exert a physiological influence on urate handling, although whether and how GLUT2 is involved in the direct regulation of serum urate levels should be explored in future studies. Additionally, among the significant loci reported (Table 1), non-synonymous variations were not found but there were intronic variations, which may influence the expression and/or splicing of transcriptional products of *GLUT2*.

**Table 1:** Previously identified genetic variations in *SLC2A2* significantly associated with gout or serum urate

Lead SNP^a^ and effect allele	Alleles	Position on Chr3^b^	Gene	Consequence from dbSNP	*p*-value	LogBF	Sex	Ancestry (GWAS lead SNP identified in)	References
With gout									
rs11711206-A	A>G	171015156	*SLC2A2*	Intron variant	1.02 × 10^−9^	-	Male	European	Major *et al.* (1)
rs35297160-C	C>G	171021192	*SLC2A2*	Intron variant	3.94 × 10^−12^	-	Full	European	Major *et al.* (1)
rs6806926-A^c^	T>A	171025958	*SLC2A2*	Intron variant	-	11.27	Full	Trans-Ancestry	Major *et al.* (1)
rs5394-A	G>A	171027104	*SLC2A2*	2kb upstream variant	-	8.36	Male	Trans-Ancestry	Major *et al.* (1)
With serum urate									
rs10513688-A	G>A	171009429	*SLC2A2*	Intron variant	1.86 × 10^−12^^d^	n/a	Full	Trans-Ancestry	Sakaue *et al.* (4)
rs9837316-T^e^	G>A/T	171033108	*SLC2A2*–*TNIK*	None	4.40 × 10^−9^	-	Full	East Asian	Sakaue *et al.* (4)

SNP, single-nucleotide polymorphism; Chr, chromosome; LogBF, Log_10_ Bayes factor for trans-ancestry GWAS; n/a, not available.

a dbSNP rs number.

b Positions of each lead SNP were based on the human genome reference assembly GRCh38.p14 (GRCh38).

c Regional plots are shown in Fig. S1.

d *P*_meta_ calculated *via* cross-population meta-analyses with three biobanks.

e This allele is reportedly associated with both reduced urate and glucose levels according to PheWeb.jp (http;//pheweb.jp), including Sakaue *et al.* (4).

Among major organs, GLUT2 is expressed on the basolateral (blood side) membrane of hepatocytes, renal proximal tubular cells, and enterocytes where it is involved in the hepatic entry of glucose from circulating blood (21), glucose reuptake from urine (22), and intestinal absorption (23), respectively. As these organs play pivotal roles in urate production (liver) and elimination (kidney and intestine), the physiological impact of GLUT2-mediated urate transport in each organ is a theoretical research topic. Notably, given the pathophysiological features of renal hypouricemia, which is an inherited disorder caused by increased urinary urate excretion that results from the complete dysfunction of URAT1 or GLUT9 (8), renal urate reuptake primarily depends on the URAT1–GLUT9 axis, and apparent urate secretion from the renal epithelial cells into the blood is mostly governed by GLUT9. Thus, although it functions as expected, the latent contribution of GLUT2 as a urate exit mechanism in this process may not be significant. In contrast, except for ABCG2, which is a high-capacity urate exporter involved in urate excretion into the intestinal tract (20), little information is available regarding physiologically important intestinal urate transporters. This point may draw increased research interest toward the small intestine (urate handling between enterocytes and blood) than to other organs.

Here, we discuss the characteristics of GLUT2-mediated transport of its substrates. According to previous studies (5, 6, 22, 24), GLUT2 has a higher *K*_m_ value for D-glucose (*K*_m_: 15–20 mM) compared with other physiologically important glucose transporters, such as GLUT1 (*K*_m_: 1–2 mM), sodium/glucose cotransporter 1 (SGLT1) (*K*_m_: 2 mM), and SGLT2 (*K*_m_: 5 mM), which means that GLUT2 is a low-affinity glucose transporter among them. The *K*_m_ value of GLUT2 was high in relation to blood glucose (one digit mM at most), suggesting that GLUT2-mediated glucose transport is not saturated under physiological conditions. Based on the findings of this study, the same applies to GLUT2-mediated urate transport. Indeed, although no saturation kinetics were observed within the tested range of urate (Fig. 2*D*), the calculated *K*_m_ value for urate of GLUT2 was approximately 4.6 mM, which exceeded the physiological concentrations of serum urate. Regarding other urate transporters, previous studies have reported *K*_m_ values of 0.37 mM for URAT1 (11), 0.56 mM for OAT10 (12), and 1.5 mM for SLC23A1 (25), all of which are importers, and 8.24 mM for ABCG2 (26), which works only as a urate exporter (Table S1 with appropriate references). For GLUT members that accept urate as substrate, the value of GLUT12 could not be determined due to observed linearity in its urate transport activities on urate concentrations (10). A previous study reported that GLUT9 is a high-capacity and low-affinity urate transporter with a *K*_m_ value of 0.89 mM (9). These data indicate that GLUT2 is a low-affinity transporter of glucose and urate.

Some study limitations and future perspectives are discussed below. To date, many studies have reported conventional or conditional *Glut2* knockout (KO) mice (5, 24, 27); however, information suggesting the physiological role of GLUT2 as a urate transporter is limited. Unlike glucose, how urate acts in such models has not been examined in previous studies, since the sugar transport function of GLUT2 has long been the focus of research. Moreover, contrary to humans, rodents, including mice, harbor the *uricase* gene, which metabolizes uric acid to allantoin (19). Because of this difference in the purine metabolism pathway, the *uricase* KO (or at least its chemical KO condition) is considered necessary for accurate analyses of urate handling in model mice. To the best of our knowledge, no studies have been conducted on *Glut2* and *uricase* double KO mice, warranting exploration in future studies.

In humans, the genetic loss-of-function of GLUT2 results in Fanconi–Bickel syndrome (FBS), a rare autosomal recessive genetic disorder of carbohydrate metabolism to which dysglycemia is associated (28, 29). FBS is characterized by the abnormal hepatorenal accumulation of glycogen, impaired glucose and galactose utilization, and proximal renal tubular dysfunction. In addition, hypouricemia and grossly increased renal urate clearance are observed in patients with FBS, suggesting severely impaired urate reabsorption from the urine in such cases (30); however, these phenotypic features seem to not directly reflect the effects of the lack of GLUT2-mediated urate transport on urate handling but result from the renal dysfunction, considering the URAT1–GLUT9 axis present in proximal tubular cells as the primary route of urate reuptake. Thus, the clinical data of subjects completely lacking GLUT2 function should be interpreted with caution. Regarding this point, while there is little clinical information available, the investigation of family members who are heterozygous for a dysfunctional *GLUT2* mutation, rather than the proband, could serve as a starting point for further research.

In summary, we revealed that GLUT2 is a bi-directional urate transporter. The following points warrant mention. Among gout- or serum urate-associated single nucleotide polymorphisms (SNPs) previously identified in *SLC2A2*, including a lead SNP rs35297160 (1), no significant signals corresponding to non-synonymous variants were detected. Thus, this study could not examine the effects of such genetic variations on cellular GLUT2 functions. Intriguingly, a GWAS reported an association between *SLC2A2* and blood glucose levels while the influence was not large (4). Despite the unclear interaction between serum urate and blood glucose levels, under the assumption that these two biochemical parameters are independent of each other, our results support the physiological impact of GLUT2-mediated urate transport. Our findings contribute to a deeper understanding of urate-handling systems in the body. Nonetheless, further studies are required to elucidate the physiological role of GLUT2 as a urate transporter.

## Experimental procedures

### Materials

The critical materials and resources used in this study are summarized in Table S2. All other chemicals used were of analytical grade and are commercially available.

### Biochemical investigations including functional assay using 293A cells transiently expressing GLUT2

Details of the Methods, including plasmid construction, cell culture, whole cell lysate preparation and immunoblotting, confocal laser scanning microscopy, and transport assays, are described in Supplementary information. All cell-based experiments were performed using human embryonic kidney 293 (HEK293)-derived 293A cell. To examine the transport activities of GLUT2 for tested substances including radiolabeled [8-^14^C]-uric acid (American Radiolabeled Chemicals), uptake assays using GLUT2-expressing 293A cells were conducted; transport activity was calculated as the incorporated clearance (μL/mg protein/min): (incorporated level of radiolabeled substance [mol/mg protein/min]/its level in the incubation mixture [mol/μl]), except for the investigation of concentration dependency. To determine the urate efflux activity of GLUT2, urate efflux assays using 293A cells co-expressing GLUT2 and SVCT2 were conducted, as described previously (18). SVCT2, a sodium-dependent urate importer, was used to sufficiently incorporate radiolabeled urate into the cells.

### Statistical analysis

All statistical analyses were performed using Excel 2019 with the Statcel4 add-in software (OMS publishing, Saitama, Japan). Different statistical tests were used for the different experiments, as described in the figure legends. When analyzing multiple groups, the homogeneity of variance among groups was confirmed using Bartlett’s test. When passing the test for homogeneity of variance, a parametric Tukey–Kramer multiple-comparison test for all pairwise comparisons or Dunnett's test for comparisons with a control group was used. For a single pair of quantitative data, after comparing the variances of a set of data using an *F*-test, an unpaired Student's *t* test or Welch's *t* test was performed. Two-factor repeated measures ANOVA was used for the time-dependent efflux assay. Statistical significance was set at *p* values < 0.05 or *p* values < 0.01. The numbers of biological replicates (*n*) are indicated in the figure legends.

## Data availability

Data are available from the corresponding authors upon reasonable request. All data relevant to the study are included in the article or uploaded as online supporting information.

## Supporting information

This article contains supporting information (1, 4, 9, 10, 11, 12, 13, 14, 16, 18, 25, 26, 31, 32, 33, 34, 35, 36).

## Conflict of interest

The authors declare that they have no conflicts of interest with the contents of this article.

## Supporting information

Mike Shipston
