# Supplementary material for: GLUT2/SLC2A2 is a bi-directional urate transporter
Source: J Biol Chem. 2025 Apr 8;301(5):108485. doi: 10.1016/j.jbc.2025.108485 (PMC12143617; doi:10.1016/j.jbc.2025.108485)
Supplement: Mike Shipston [file mmc1.docx]

**Supplementary Information**

**GLUT2/SLC2A2 is a bi-directional urate transporter**

Yu Toyoda^1,2^, Ryuichiro Shigesawa^2^, Tony R. Merriman^3^, Hirotaka Matsuo^1,*^, Tappei Takada^2,*^

1. Department of Integrative Physiology and Bio-Nano Medicine, National Defense Medical College, 3-2 Namiki, Tokorozawa, Saitama 359-8513, Japan
2. Department of Pharmacy, The University of Tokyo Hospital, 7-3-1 Hongo, Bunkyo-ku, Tokyo 113-8655, Japan
3. Division of Clinical Immunology and Rheumatology, University of Alabama at Birmingham, Birmingham, AL 35233, USA

^*^ **Correspondence to:** matsuo29@gmail.com (HM); tappei-tky@g.ecc.u-tokyo.ac.jp (TT)

**SUPPLEMENTARY METHODS**

**SUPPLEMENTARY FIGURES S1–S4**

**SUPPLEMENTARY TABLES S1–S3**

**SUPPLEMENTARY REFERENCES**

**SUPPLEMENTARY METHODS**

***Materials***

The critical materials and resources used in this study are summarized in **Table S2**. All other chemicals used were of analytical grade and are commercially available. To prepare stock solutions, authentic compounds for GLUT2 inhibition assay were dissolved with dimethyl sulfoxide (DMSO; Nacalai Tesque, Kyoto, Japan).

***Plasmid constructions***

Full-length of the wild-type (WT) human GLUT2/SLC2A2 (National Center for Biotechnology Information [NCBI] accession no. NM_000340.2) open reading frame (ORF) was PCR amplified from a total cDNA library of human hepatoma HepG2 cells prepared in our previous study (1). After cloning into a pGEM-T Easy vector (Promega, Fitchburg, WI, USA), the ORF was inserted into a pmRFP-C1 vector plasmid, which was obtained by replacing the enhanced green fluorescence protein (EGFP) ORF in the pEGFP-C1 vector (Clontech Laboratories, Palo Alto, CA, USA) with the monomeric red fluorescence protein (mRFP) ORF amplified from a pCAG-mRFP vector used in our previous study (2). The resulting construct was used to express the mRFP-tagged GLUT2 (mRFP-GLUT2). To remove the mRFP ORF for the expression of GLUT2 without an mRFP tag, site-directed mutagenesis was successfully employed. SVCT2/SLC23A2 WT ORF (NCBI accession no. NM_005116) inserted into the pEGFP-N1 vector and ABCG2 WT ORF (NCBI accession no. NM_004827) into the pEGFP-C1 vector were obtained from a previous study (3). All plasmid constructs were confirmed via full sequencing using a BigDye Terminator v3.1 (Applied Biosystems, Foster City, CA, USA) on an Applied Biosystems 3130 Genetic Analyzer (Applied Biosystems), according to the manufacturer’s protocol. All the plasmids used in each experiment were obtained from the same lot.

***Cell culture***

Human embryonic kidney 293 (HEK293)-derived 293A cells were maintained in Dulbecco’s Modified Eagle’s Medium (Nacalai Tesque) supplemented with 10% fetal bovine serum (Cosmo Bio, Tokyo, Japan), 1% penicillin-streptomycin (Nacalai Tesque), 2 mM L-glutamine (Nacalai Tesque), and MEM Non-Essential Amino Acids (Life Technologies, Tokyo, Japan) at 37°C in a humidified atmosphere of 5% (v/v) CO_2_ in air, as described previously (4). All experiments were performed using 293A cells from passages 13–20.

Vector plasmids encoding each construct or mock (empty pmRFP-C1 vector) were transfected into 293A cells using polyethyleneimine “MAX” (PEI-MAX) (Polysciences, Warrington, PA, USA) as described previously (5), with some minor modifications. Briefly, for *in vitro* assays, each vector plasmid was transfected into 293A cells on 12-well cell culture plates 24 h after seeding (0.92 × 10^5^ cells/cm^2^) with a forward transfection approach. For microscopic observation, we employed a reverse transfection approach in which freshly harvested 293A cells (0.75 × 10^6^ cells/dish) were treated with the transfection mixture and then re-seeded onto a collagen-coated glass-bottom dish (Matsunami Glass, Tokyo, Japan). The amount of plasmid DNA used for transfection was adjusted among the sample groups, and the medium was replaced with a fresh medium after the first 24 h of incubation.

***Whole cell lysate preparation and immunoblotting***

Forty-eight hours after plasmid transfection, the cells were washed with ice-cold phosphate-buffered saline without potassium [PBS (−)] twice and were lysed with an ice-cold lysis buffer A (50 mM Tris/HCl [pH 7.4], 1 mM dithiothreitol, 1% [w/v] Triton X-100, and cOmplete, EDTA-free protease inhibitor cocktail [Roche, Basel, Switzerland]). Whole-cell lysates (WCLs) were prepared as described previously (5). The protein concentration of WCLs was quantified using a Pierce BCA Protein Assay Kit (Thermo Fisher Scientific, Carlsbad, CA, USA) according to the manufacturer’s protocol, with bovine serum albumin (BSA) as the standard.

Immunoblot analyses were performed as previously described (5), with minor modifications. Briefly, the WCLs were separated via sodium dodecyl sulfate polyacrylamide gel electrophoresis and transferred onto an Immobilon-P polyvinylidene difluoride membrane (Millipore, Bedford, MA, USA) via electroblotting at 15 V for 60 min. The membranes were blocked via incubation in Tris-buffered saline containing 0.05% Tween 20 and 5% skim milk. Blots were probed with the appropriate antibodies (**Table S2**), and the signals were visualized using chemiluminescence and detected using a multi-imaging analyzer Fusion Solo 4 system (Vilber Lourmat, Eberhardzell, Germany).

***Confocal laser scanning microscopy***

Specimens were prepared as previously described (6), with minor modifications. Briefly, 48 h after transfection, cells were fixed with ice-cold 4% paraformaldehyde phosphate buffer solution (FUJIFILM Wako Pure Chemical, Osaka, Japan) for 10 min at room temperature. After washing with PBS (–), the cells were treated with Hoechst 33342 (Thermo Fisher Scientific) [final concentration: 5 μg/mL in PBS (–)] in the dark for 10 min at room temperature. After visualizing the nuclei, the cells were washed twice with PBS (–) and mounted on VECTASHIELD Mounting Medium (Vector Laboratories, Burlingame, CA, USA). Fluorescence was observed using an FV10i Confocal Laser Scanning Microscope (Olympus, Tokyo, Japan) to analyze the localization of mRFP-fused GLUT2.

***Transport assay using 293A cells transiently GLUT2-expressing***

To examine the transport activities of GLUT2 for urate, 2-deoxy-D-glucose (2-DG), or fructose, uptake assays using GLUT2-expressing 293A cells were conducted as described in our previous studies (2,3), with some modifications. Briefly, 48 h after plasmid transfection, the cells were washed twice with the indicated transport (TP) buffer (the composition of each transport buffer is summarized in **Table S3**) and preincubated in TP buffer at 37°C for 10 min. Then, the TP buffer was replaced with prewarmed fresh TP buffer containing the corresponding radiolabeled substances, [8-^14^C]-uric acid (American Radiolabeled Chemicals, St. Louis, MO, USA), [1,2-^3^H(N)]-2-DG (Moravec, Brea, CA, USA), or [^3^H(G)]-fructose (American Radiolabeled Chemicals), at the indicated concentrations, and the cells were further incubated for the indicated periods. Unless otherwise noted, glucose- and glutamine-free Krebs–Ringer buffer was employed for uptake incubation. The [8-^14^C]-uric acid concentration and incubation period in the present study were 10 μM and 1 min, respectively. For inhibition assays, 0.5% DMSO was used as a vehicle control, except for the investigation of the effect of glucose (Nacalai Tesque) or uric acid (FUJIFILM Wako Pure Chemical) on GLUT2 function. Subsequently, the cells were washed thrice with ice-cold TP buffer and then lysed with 750 μL of 0.4 M NaOH on ice with gentle shaking for 1 h. The lysates were neutralized with 150 μL of 2 M HCl. Radioactivity in the lysate was measured using a liquid scintillator (Tri-Carb 3110TR; PerkinElmer, Waltham, MA, USA), and protein concentrations in the lysates were determined using the Pierce BCA Protein Assay Kit, as described above.

Transport activity was calculated using the formula: incorporated clearance (μL/mg protein/min) = (incorporated level of radiolabeled substance [disintegration per minute (DPM, an indicator for the amount of the substance)/mg protein/min] / radiolabeled substance level in the incubation mixture [DPM/μL]). GLUT2-mediated transport activity was calculated by subtracting the transport activity of mock cells from that of GLUT2-expressing cells. To determine the kinetic parameters (Michaelis–Menten constant [*K*_m_] and maximal velocity [*V*_max_]) of GLUT2-mediated urate transport, the Michaelis–Menten model was fitted to the experimental transport rates and urate concentrations using nonlinear regression curve fitting with GraphPad Prism 8 (GraphPad Software, San Diego, CA, USA).

***Urate efflux assay using GLUT2- and SVCT2 co-expressing 293A cells***

To determine the urate efflux activity of GLUT2, urate efflux assays using 293A cells co-expressing GLUT2 and SVCT2 were conducted in 24-well cell culture plates, as described previously (3). A mock plasmid was used to equalize the amount of plasmid used for transient transfection among the wells. SVCT2, a sodium-dependent urate importer, was used to sufficiently incorporate radiolabeled urate into the cells, and ABCG2, a urate exporter, was used as a positive control for the assay.

The experimental flow of efflux assay was comprised of the urate accumulation and secretion steps (**Fig. S4*A***). For the accumulation step, 48 h after double plasmid transfection (total of 1 μg: 0.5 μg of SVCT2/pEGFP-N1 and 0.5 μg of GLUT2/pmRFP-C1 or empty pmRFP-C1), the cells were washed twice with Krebs–Ringer buffer and preincubated in the same buffer at 37°C for 10 min. The buffer was then replaced with fresh prewarmed Krebs–Ringer buffer containing 10 μM [8-^14^C]-urate, and the cells were incubated at 37°C for 40 min to incorporate radiolabeled urate.

For the secretion step, the cells were washed twice on ice with ice-cold Na^+^-, glucose-, and glutamine-free Krebs–Ringer buffer (efflux TP buffer) containing 5 μM non-radiolabeled urate at this time point to remove the remaining extracellular radiolabeled urate. The buffer was then replaced with 500 μL of the prewarmed efflux TP buffer (time, 0 min), and the cells were incubated at 37°C for 75 min. At specific timepoints (5, 15, 30, and 45 min), 100 μL of the efflux TP buffer was collected and an equal volume of fresh prewarmed efflux TP buffer was added to maintain the total volume in each well. At the end of the secretion step (75 min), the efflux TP buffer was collected and the cells were washed twice with ice-cold efflux TP buffer on ice. As described above, cell lysates were prepared and subjected to protein concentration determination; radioactivity in the lysates and collected buffers was measured using the liquid scintillator (Tri-Carb 3110TR).

Cellular urate efflux was evaluated as the apparent urate efflux activity and secreted/remaining proportion as previously described (3). These indicators were defined and calculated as follows: apparent urate efflux activity [mg protein/min] = [8-^14^C]-urate efflux rate (0–5 min) [DPM/ min]/cellular [8-^14^C]-urate level at 0 min [DPM/mg protein], and the activity was expressed as a percentage of control; secreted proportion [%] = net amount of media-released [8-^14^C]-urate at indicated time point per well [DPM]/initial amount of cellular [8-^14^C]-urate at 0 min per well [DPM] × 100. To calculate the initial amount of cellular [8-^14^C]-urate at 0 min, the intracellular radioactivity at the endpoint and the total amount of media-released radioactivity during the secretion step were summed. The remaining proportion was calculated as by subtracting the secreted proportion [%] from 100.

**SUPPLEMENTARY FIGURES**

**Fig. S1. Regional association plot showing the association between *SLC2A2* and gout.**

Data and image were obtained from a previous study (7).

**Fig. S2. Effects of glucose on GLUT2-mediated urate transport, suggesting that physiologically relevant levels of glucose would hardly affect the GLUT2-mediated urate transport.**

The corresponding cellular urate transport activities are shown in **Fig. 2*E***. ^**^, *p* < 0.01; ns, not significantly different between the indicated groups [Dunnett’s test (*vs.* control)].

**Fig. S3. Effects of glucose on cellular fructose transport activities.**

Using 293A cells transiently expressing GLUT2 48 h after plasmid transfection, uptake assays were conducted in glucose- and glutamine-free Krebs–Ringer buffer at pH 7.4 for 1 min. The concentrations of [^3^H(G)]-fructose in the transport buffer was 100 nM. Mock, control transfected with empty vector (pmRFP-C1 plasmid without insert). Data are expressed as the mean ± SD; *n* = 3–4.

**Fig. S4. GLUT2 functions as a urate exporter.**

HEK293-derived 293A cells transiently co-expressing GLUT2 and SVCT2 were used 48 h after plasmid transfection. ABCG2 (urate exporter), positive control. The details are described in **Supplementary Methods**. UA, urate. ***A***, Schematic illustration of the [8-^14^C]-urate efflux assay. ***B***, [8-^14^C]-Urate levels in cells at the beginning and end of the efflux phase (0–75 min). ***C***, Apparent urate efflux activity. The activities are expressed as a percentage of the control. Data are expressed as the mean ± SD; where the vertical bars are not visible, the SD was contained within the limits of the symbol; *n* = 4. ^*^, *p* < 0.05; ^**^, *p* < 0.01; ^##^, *p* < 0.01 (Dunnett’s test among all the groups indicated).

**SUPPLEMENTARY TABLES**

**Table S1.** **Experimentally determined values of Michaelis-Menten constant (*K*_m_) for urate of human urate transporters.**

| **Urate transporters** | | ***K*_m_ for urate [μM]** | **References** |
| --- | --- | --- | --- |
| *SLC2A family proteins* | |  |  |
|  | GLUT9/SLC2A9 | 890; 981 | Vitart *et al.*, 2008 (8); Caulfield *et al.*, 2008 (9) |
|  | GLUT2/SLC2A2 | 4,583 | This paper |
|  | GLUT12/SLC2A12 | N.D. | Toyoda *et al.*, 2020 (2) |
|  |  |  |  |
| *SLC22A family proteins* | |  |  |
|  | URAT1/SLC22A12 | 371 | Enomoto *et al*., 2002 (10) |
|  | OAT10/SLC22A13 | 558 | Toyoda *et al.*, 2022 (5) |
|  | OAT1/SLC22A6 | 943 | Ichida *et al.*, 2003 (11) |
|  | OAT2/SLC22A7 | 1,168 | Sato *et al.*, 2010 (12) |
|  | OAT3/SLC22A8 | 2,888 | Kimura *et al.*, 2000 (13) |
|  | OAT4/SLC22A11 | 3,780 | Kimura *et al.*, 2001 (14) |
|  |  |  |  |
| *SLC23A family proteins* | |  |  |
|  | SVCT1/SLC23A1 | 1,511 | Toyoda *et al.*, 2023 (15) |
|  | SVCT2/SLC23A2 | 3,860 | Toyoda *et al.*, 2023 (3) |
|  |  |  |  |
| *ABC proteins* | |  |  |
|  | ABCC4 | 1,550 | Van Aubel et al., 2005 (16) |
|  | ABCG2 | 8,240 | Matsuo *et al.*, 2009 (17) |

URAT, urate transporter; OAT, organic anion transporter; GLUT, glucose transporter; ABC, ATP-binding cassette;

N.D., not determined due to the observed linear increase in the concentration-dependent urate transport within the tested range.

**Table S2. Key resources.**

| **REAGENT or RESOURCE** | **SOURCE** | **IDENTIFIER** |
| --- | --- | --- |
| ***Antibodies*** | | |
| RFP Monoclonal Antibody (mouse mAb) | MBL International | Cat# M155-3; RRID: AB_1278880; 1:1,000 dilution |
| Rabbit polyclonal anti-α-tubulin | Abcam | Cat# ab15246; RRID: AB_301787  1:1,000 dilution |
| Donkey anti-rabbit IgG-horseradish peroxidase (HRP)-conjugate | GE Healthcare | Cat# NA934V; RRID: AB_772206; 1:3,000 dilution |
| Sheep anti-mouse IgG-horseradish peroxidase antibody | GE Healthcare | Cat# NA931; RRID: AB_772210;  1:3,000 dilution |
| ***Chemicals*** | | |
| 2-Deoxy-D-glucose, [1,2-^3^H(N)]- (30 Ci/mmol) | Moravek | Cat# MT-911 |
| Fructose, D-[^3^H(G)] (5 Ci/mmol) | American Radiolabeled Chemicals | Cat# ART0329 |
| [8-^14^C]-Uric acid (53 mCi/mmol) | American Radiolabeled Chemicals | Cat# ARC0513 |
| Uric acid | FUJIFILM Wako Pure Chemical | Cat# 210-00225; CAS: 69-93-2 |
| D-(+)-Glucose | Nacalai Tesque | Cat# 16806-25; CAS: 50-99-7 |
| Polyethelenimine “MAX” (PEI-MAX) | Polysciences | Cat# 24765; CAS: 49553-93-7 |
| Clear-sol II | Nacalai Tesque | Cat# 09136-83 |
| Hoechst 33342, Trihydrochloride, Trihydrate | Thermo Fisher Scientific | Cat# H1399 |
| BAY-876 | Sigma-Aldrich | Cat# SML1774-5MG; CAS: 1799753-84-6 |
| Benzbromarone | FUJIFILM Wako Pure Chemical | Cat# 028-15851; CAS: 3562-84-3 |
| Cytochalasin B | FUJIFILM Wako Pure Chemical | Cat# 034-17554; CAS: 14930-96-2 |
| Forskolin | Tokyo Chemical Industry | Cat# F0855; CAS: 66575-29-9 |
| Glutor | Sigma-Aldrich | Cat# SML2765-25MG; CAS: 2561471-22-3 |
| Phloretin | Abcam | Cat# ab143143; CAS: 60-82-2 |
| WZB117 | Adipogen Life Sciences | Cat# AG-CR1-3694-M005; CAS: 1223397-11-2 |
| ***Critical Commercial Assays*** | | |
| Pierce^TM^ BCA Protein Assay Reagent A & B | Thermo Fisher Scientific | Cat# 23223, Cat# 23224 |
| PureLink HiPure Plasmid Filter Midiprep Kit | Thermo Fisher Scientific | Cat# K210015 |
| ***Recombinant DNA*** | | |
| The complete human GLUT2 cDNA | This paper | NCBI Ref Sequence: NM_000340.2 |
| The complete human SVCT2 cDNA | Toyoda et al., 2023 (3) | NCBI Ref Sequence: NM_005116 |
| The complete human ABCG2 cDNA | Toyoda et al., 2023 (3) | NCBI Ref Sequence: NM_004827 |
| pCAG-mRFP | Toyoda et al., 2020 (2) | N/A |
| pmRFP-C1 | This paper | N/A |
| ***Experimental Models: Cell Lines*** | | |
| Human: 293A cells | Invitrogen | R70507 |
| Human: HepG2 cells | Miyata et al., 2022, (1) | N/A |
| ***Deposited data*** | | |
| GWAS summary statics of a previous study | Sakaue et al., 2021, (18) | https://doi.org/10.1038/s41588-021-00931-x; https://pheweb.jp/ |
| Results of single-ancestry and trans-ancestry meta-analyses | Major et al., 2024, (7) | https://www.nature.com/articles/s41588-024-01921-5 |
| ***Software and Algorithms*** | | |
| Excel 2019 | Microsoft | https://products.office.com/ja-jp/home |
| Statcel4 add-in software | OMS Publishing | http://www.oms-publ.co.jp/ |
| GraphPad Prism 8 | GraphPad Software | https://www.graphpad.com/ |

**Table S3. Compositions of transport buffers used in the present study.**

|  | **Final concentrations [mM]** |
| --- | --- |
| ***Glc- and Gln-free Krebs–Ringer buffer*** |  |
| NaCl | 133 |
| KCl | 4.93 |
| MgSO_4_ | 1.23 |
| CaCl_2_ | 0.85 |
| HEPES | 10 |
| MES | 10 |
|  | pH 6.4, 7.4 |
|  |  |
| ***Na^+^-, Glc- and Gln-free Krebs–Ringer buffer*** |  |
| Choline-Cl | 133 |
| KCl | 4.93 |
| MgSO_4_ | 1.23 |
| CaCl_2_ | 0.85 |
| HEPES | 10 |
| MES | 10 |
|  | pH 7.4 |
|  |  |
| ***K^+^-high buffer Glc-free*** |  |
| NaCl | None |
| KCl | 145.4 |
| MgSO_4_ | 0.8 |
| CaCl_2_ | 1.8 |
| HEPES | 25 |
| Tris | 25 |
|  | pH 7.4 |
|  |  |
| ***Krebs–Ringer buffer*** |  |
| NaCl | 133 |
| KCl | 4.93 |
| MgSO_4_ | 1.23 |
| CaCl_2_ | 0.85 |
| HEPES | 10 |
| MES | 10 |
| D-Glucose | 5 |
| L-Glutamine | 5 |
|  | pH 7.4 |

*NaCl was replaced with choline-Cl for Na^+^-free Krebs–Ringer buffer.

Glc, glucose; Gln, glutamine.

**SUPPLEMENTARY REFERENCES**

1. Miyata, H., Toyoda, Y., Takada, T., Hiragi, T., Kubota, Y., Shigesawa, R.*, et al.* (2022) Identification of an exporter that regulates vitamin C supply from blood to the brain. *iScience* **25**, 103642

2. Toyoda, Y., Takada, T., Miyata, H., Matsuo, H., Kassai, H., Nakao, K.*, et al.* (2020) Identification of GLUT12/SLC2A12 as a urate transporter that regulates the blood urate level in hyperuricemia model mice. *Proc. Natl. Acad. Sci. U. S. A.* **117**, 18175-18177

3. Toyoda, Y., Miyata, H., Shigesawa, R., Matsuo, H., Suzuki, H., and Takada, T. (2023) SVCT2/SLC23A2 is a sodium-dependent urate transporter: functional properties and practical application. *J. Biol. Chem.* **299**, 104976

4. Higashino, T., Morimoto, K., Nakaoka, H., Toyoda, Y., Kawamura, Y., Shimizu, S.*, et al.* (2020) Dysfunctional missense variant of OAT10/SLC22A13 decreases gout risk and serum uric acid levels. *Ann. Rheum. Dis.* **79**, 164-166

5. Toyoda, Y., Kawamura, Y., Nakayama, A., Morimoto, K., Shimizu, S., Tanahashi, Y.*, et al.* (2022) OAT10/SLC22A13 Acts as a Renal Urate Re-Absorber: Clinico-Genetic and Functional Analyses With Pharmacological Impacts. *Front. Pharmacol.* **13**, 842717

6. Toyoda, Y., Takada, T., Saito, H., Hirata, H., Ota-Kontani, A., Kobayashi, N.*, et al.* (2020) Inhibitory effect of Citrus flavonoids on the in vitro transport activity of human urate transporter 1 (URAT1/SLC22A12), a renal re-absorber of urate. *NPJ Sci Food* **4**, 3

7. Major, T. J., Takei, R., Matsuo, H., Leask, M. P., Sumpter, N. A., Topless, R. K.*, et al.* (2024) A genome-wide association analysis reveals new pathogenic pathways in gout. *Nat. Genet.* **56**, 2392-2406

8. Vitart, V., Rudan, I., Hayward, C., Gray, N. K., Floyd, J., Palmer, C. N.*, et al.* (2008) SLC2A9 is a newly identified urate transporter influencing serum urate concentration, urate excretion and gout. *Nat. Genet.* **40**, 437-442

9. Caulfield, M. J., Munroe, P. B., O'Neill, D., Witkowska, K., Charchar, F. J., Doblado, M.*, et al.* (2008) SLC2A9 is a high-capacity urate transporter in humans. *PLoS Med.* **5**, e197

10. Enomoto, A., Kimura, H., Chairoungdua, A., Shigeta, Y., Jutabha, P., Cha, S. H.*, et al.* (2002) Molecular identification of a renal urate anion exchanger that regulates blood urate levels. *Nature* **417**, 447-452

11. Ichida, K., Hosoyamada, M., Kimura, H., Takeda, M., Utsunomiya, Y., Hosoya, T.*, et al.* (2003) Urate transport via human PAH transporter hOAT1 and its gene structure. *Kidney Int.* **63**, 143-155

12. Sato, M., Mamada, H., Anzai, N., Shirasaka, Y., Nakanishi, T., and Tamai, I. (2010) Renal secretion of uric acid by organic anion transporter 2 (OAT2/SLC22A7) in human. *Biol. Pharm. Bull.* **33**, 498-503

13. Kimura, H., Ichida, K., Hosoyamada, M., Oono, I., Endou, H., and Hosoya, T. (2000) Analysis of uric acid transport via hOAT3. *Gout and Nucleic Acid Metabolism* **24**, 115-121

14. Kimura, H., Ichida, K., Hosoyamada, M., Oono, I., Endou, H., and Hosoya, T. (2001) Urate transport via hOAT4. *Gout and Nucleic Acid Metabolism* **25**, 113-120

15. Toyoda, Y., Miyata, H., Uchida, N., Morimoto, K., Shigesawa, R., Kassai, H.*, et al.* (2023) Vitamin C transporter SVCT1 serves a physiological role as a urate importer: functional analyses and in vivo investigations. *Pflugers Arch.* **475**, 489-504

16. Van Aubel, R. A., Smeets, P. H., van den Heuvel, J. J., and Russel, F. G. (2005) Human organic anion transporter MRP4 (ABCC4) is an efflux pump for the purine end metabolite urate with multiple allosteric substrate binding sites. *Am. J. Physiol. Renal Physiol.* **288**, F327-333

17. Matsuo, H., Takada, T., Ichida, K., Nakamura, T., Nakayama, A., Ikebuchi, Y.*, et al.* (2009) Common defects of ABCG2, a high-capacity urate exporter, cause gout: a function-based genetic analysis in a Japanese population. *Sci. Transl. Med.* **1**, 5ra11

18. Sakaue, S., Kanai, M., Tanigawa, Y., Karjalainen, J., Kurki, M., Koshiba, S.*, et al.* (2021) A cross-population atlas of genetic associations for 220 human phenotypes. *Nat. Genet.* **53**, 1415-1424
